# Outcome after Thrombectomy of Acute M1 and Carotid-T Occlusions with Involvement of the Corticospinal Tract in Postinterventional Imaging

**DOI:** 10.3390/jcm11102823

**Published:** 2022-05-17

**Authors:** Sarah Christina Reitz, Ellen Gerhard, Stella Breuer, Ferdinand Oliver Bohmann, Waltraud Pfeilschifter, Joachim Berkefeld

**Affiliations:** 1 Department of Neurology, University Hospital Frankfurt, Goethe University, 60528 Frankfurt am Main, Germany; ferdinand.bohmann@kgu.de (F.O.B.); waltraud.pfeilschifter@klinikum-lueneburg.de (W.P.); 2 Institute of Neuroradiology, University Hospital Frankfurt, Goethe University, 60528 Frankfurt am Main, Germany; ellen.gerhard@web.de (E.G.); stella.breuer@kgu.de (S.B.); joachimberkefeld@icloud.com (J.B.); 3 Klinik für Neurologie und Klinische Neurophysiologie, Klinikum Lüneburg, 21339 Lüneburg, Germany

**Keywords:** corticospinal tracts, acute stroke, cerebrovascular disease, endovascular recanalization, endovascular treatment

## Abstract

Objectives: Until now, thrombectomy studies have provided little reliable information about the correlation between the infarct topography and clinical outcome of acute stroke patients with embolic large-vessel occlusions. Therefore, we aimed to analyze whether infarcts of the corticospinal tracts in the central white matter (CWM) or the internal capsule on postinterventional imaging controls are associated with poor clinical outcome after thrombectomy. Materials and Methods: We retrospectively analyzed imaging data from 70 patients who underwent endovascular thrombectomy for emergent middle cerebral artery or carotid-T occlusions. Inclusion criteria were postinterventional infarct demarcation in the regions of the internal capsule, caudate, lentiform nucleus, and CWM. Primary outcome was the mRS after 90 days and secondary endpoints were subgroup analyses regarding additional cortical infarction. Conclusions: In this exploratory study, we found no indication that infarcts in the course of the corticospinal tracts predict poor clinical outcome after successful thrombectomy in patients with embolic carotid-T or M1 occlusions. In our analysis, a significant number of patients showed a favorable 90 day outcome. Additional cortical infarcts may have a greater impact on the risk of an unfavorable outcome. Results: Good clinical outcome after 90 days (mRS 0–2) was shown in 36 out of 70 patients (51.4%), with excellent clinical outcome (mRS 0–1) in 23 patients (32.9%). Here, 58.6% patients lived at home without nursing service after 90 days. Patients with minimal additional cortical infarction in postinterventional imaging had a 75.6% better chance of excellent outcome.

## 1. Introduction

Over the past years, endovascular thrombectomy (EVT) with a stent retriever in combination with thrombus aspiration has become the standard of care for acute stroke secondary to large-vessel occlusion (LVO), showing reductions in neurological deficits and improvements in patient outcome. Randomized studies have consistently shown the statistically significant superiority of mechanical thrombectomy with stent retrievers over intravenous thrombolysis with a recombinant tissue plasminogen activator (rtPA) (e.g., MR CLEAN [1], ESCAPE [2], REVASCAT [3], SWIFTPRIME [4], EXTEND-IA [5]).

To assess the extent of early ischemic changes on brain imaging for acute stroke treatment, the Alberta Stroke Program Early Computed Tomography Score (ASPECTS) [6] is used in clinical practice. The prediction of functional recovery is challenging due to the high interindividual variability [7].

A recent subgroup analysis of the HERMES meta-analysis suggested better outcomes at 90 days than standard medical therapy across a broad range of baseline imaging categories, including infarcts affecting more than 33% of the middle cerebral artery (MCA) territory or ASPECTS values of less than 6 [8]. Trials with extended time windows up to 24 h have used infarct volumes (DAWN [9]) or perfusion imaging (EXTEND-IA [5]) as inclusion criteria for the definition of salvageable brain tissue. However, the influence of infarct topography and the involvement of functional important structures has not been studied in detail. The integrity of the corticospinal tract has already proven to be a factor for motor outcome [10]. The ASPECTS covers only the internal capsule and the basal ganglia but not the central corona radiata in the deep white matter. Due to the arterial supply from the poorly collateralized ascending perforating branches, the deep white matter tracts are frequently involved in stroke patients with embolic LVO of the carotid-T or the M1-segment. White matter involvement has been described as an independent negative prognostic marker [11]. Evidence regarding the prognostic relevance of the infarct pattern and location is inconclusive [12,13].

A retrospective study showed that isolated basal ganglia infarction in 57 patients on pretreatment DWI seemed to predict successful reperfusion after EVT in patients with occlusions in the distal intracranial internal carotid artery (ICA) or the MCA. However, isolated basal ganglia infarction was not associated with a 90 day good outcome in this patient cohort [14]. The key point may not be basal ganglia infarction but the involvement of the white matter tracts in the adjacent central corona radiata or the internal capsule. Since their main vascular supply is the lenticulostriate arteries, their tolerance for ischemia is very limited if the M1-segment is blocked by an embolus. Other recent studies have shown an association between infarct size and outcome, but only with moderate strength [15].

Our hypothesis was that demarcated infarcts in the area of the corticospinal tracts (here defined as infarctions in the regions of the internal capsule, caudate, lentiform nucleus, and CWM) on postinterventional CT or MRI controls are associated with poor clinical outcome, despite successful thrombectomy of embolic M1 or carotid-T occlusions. Therefore, we reviewed imaging studies and clinical records of these cases and compared a subgroup of patients with only involvement of corticospinal tracts with patients with additional cortical infarcts. This explorative study provides first insights into the potential role of the infarct topography regarding a potentially good outcome.

## 2. Materials and Methods

### 2.1. Study Design

This study was conducted at the University Hospital Frankfurt, an EVT-capable stroke center with 24/7 thrombectomy capacity. Data were retrieved from a mandatory prospective stroke inpatient quality assurance registry.

For the retrospective analysis, all patients who underwent thrombectomy because of acute ischemic stroke in our department from April 2016 to January 2020 were filtered for the following inclusion criteria: (1) embolic large-vessel occlusion in the anterior circulation (M1 segment of the middle cerebral artery (MCA) or occlusion of the carotid artery T); (2) infarct demarcation in the postinterventional imaging in the region of the caudate (C) and lentiform nucleus (L) ASPECTS areas and additional involvement of the white matter tracts of the internal capsule (CI) or the CWM (which is not represented in the ASPECTS). Imaging selection was performed by consensus with two experienced neuroradiologists according to postinterventional CT or MRI controls, which were routinely performed the day after the thrombectomy procedure. In addition, preinterventional imaging studies were also analyzed in the same way.

Eligible patients received intravenous thrombolysis (IVT) before EVT. Endovascular therapy was performed under general anesthesia with established techniques (stent retrievers in combination with aspiration).

**Figure 1:**
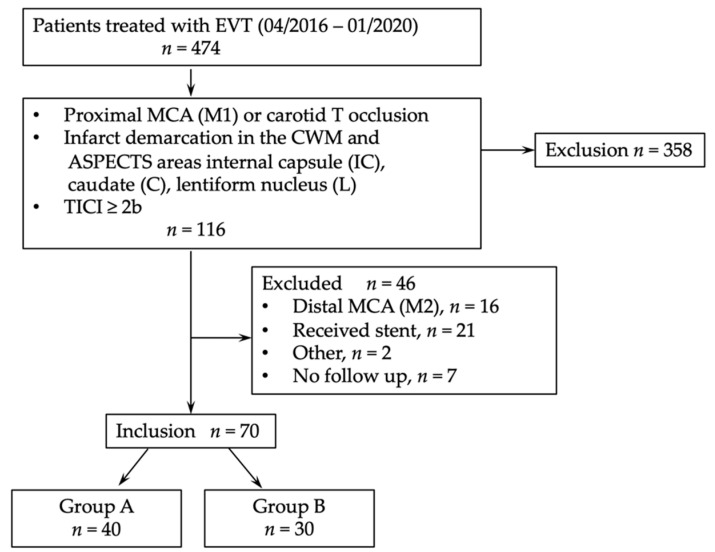
Consort diagram. EVT, endovascular thrombectomy; MCA, middle cerebral artery; TICI, thrombolysis in cerebral infarction.

Angiographic outcome was defined by the degree of revascularization at the end of the endovascular procedure using the modified Thrombolysis in Cerebral Infarction (mTICI) score [16]. Patients with atherothrombotic or distal M2 occlusions were excluded, as well as patients with an unsuccessful thrombectomy maneuver (mTICI 0-2a). For an overview, see Figure 1 (consort diagram).

**Figure 2:**
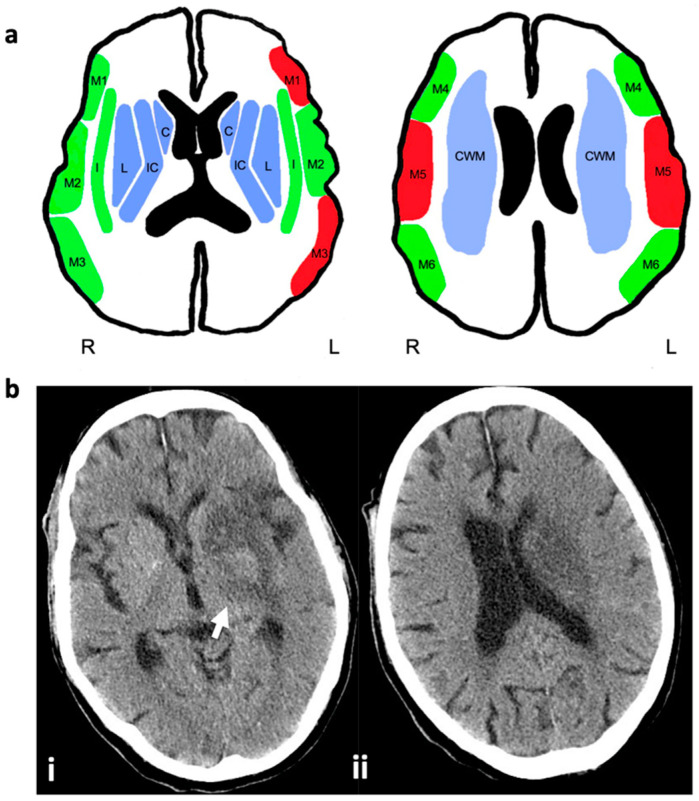
ASPECTS presentation, group definition, and case example for imaging selection criteria: (**a**) subganglionic nuclei: frontal operculum (M1), anterior temporal lobe (M2), posterior temporal lobe (M3); supraganglionic nuclei: anterior MCA (M4), lateral MCA (M5), posterior MCA (M6); basal ganglia: caudate (C), lentiform nucleus (L), insula (I), internal capsule (IC), central white matter (CWM). *Blue colored areas* correspond to inclusion criteria: infarct demarcation in the postinterventional imaging in the regions of C, L and CI or CWM (the latter is not represented in the ASPECTS). *Red colored areas* show eloquent cortical ASPECTS areas with impacts on sensomotoric or language functions (M1 and M3 left; M5 on both sides). *Green colored* areas show non-eloquent cortical areas (M1 and M3 right; M2, M4, M6 on both sides). Group A: Affection of ASPECTS areas C, L, and CI or CWM plus one green cortical area. Group B: Affection of ASPECTS areas C, L, and CI plus one red cortical area or more than one green cortical area. (**b**) CT of an 80 years old stroke patient with M1 occlusion on the left side, obtained 24 h after successful thrombectomy to illustrate imaging inclusion criteria of suspected lesion of the corticospinal tract: (i) hypodense infarct demarcation in the left caudate nucleus, basal ganglia, and posterior limb of the internal capsule (arrow); (ii) infarct of the central white matter on the left side above the level of the basal ganglia. The cortical territories of the middle cerebral artery are spared.

Eloquent areas were defined as regions with impacts on sensomotoric or language functions (M1 and M3 left side; M5 on both sides). In the next step, we divided the study population into two groups: group A, having a maximum of one affected non-eloquent cortical ASPECTS area (M1 and M3 right side; M2, M4, M6 on both sides) additional to the infarct demarcation in the CWM and ASPECTS areas CI, L, and C; group B, having additional affection of an eloquent cortical ASPECTS area (see above) or ≥2 cortical ASPECTS area (M1 and M3 right side; M2, M4, M6 on both sides). Figure 2 will give an overview of the ASPECTS presentation and group definition.

For the overall outcome, we analyzed the degree of disability on the modified Rankin Scale (mRS) at 90 days after stroke. Further analyses were performed for the mRS shift after 90 days, good clinical outcome (mRS score ≤ 2), and excellent outcome (defined as mRS ≤ 1). Additionally, the living status after 90 days was assessed (home, nursing home, nursing at home, semiresidential rehab, residential rehab, hospital, rehab, dead).

Safety endpoints were mortality, unfavorable outcome (mRS 3–5), and intracranial hemorrhage. The occurrence of intracranial hemorrhage was defined according to the Heidelberg Bleeding Classification 2015 [17]. Stroke severity was assessed using the National Institutes of Health Stroke Scale (NIHSS) at admission, 24 h after EVT, and at discharge.

The study was approved by the local ethics committee of the Goethe University Hospital Frankfurt (approval number 2021-70) and was conducted according to the principles of the Declaration of Helsinki.

### 2.2. Statistical Analysis

Regarding the baseline characteristics, the data are presented as medians (interquartile ranges, IQR) or means (depending on the presence of normal distribution, tested by quantile–quantile plots) and numbers with percentages, unless otherwise indicated. The statistical significance of differences was assessed via the Wilcoxon matched pair test, Mann–Whitney U test, or Friedmann test, depending on the scale and group. Pearson’s square test or Fisher exact test was used for binary variables. The influence of group dependency for good and excellent outcomes was evaluated using univariate binary and ordinary logistic regression regarding the dependent variable distribution. Unadjusted common odds ratios (OR) are reported with 95% confidence intervals (CI) to indicate statistical precision. The significance level was set to *p* < 0.05.

Statistical analysis was performed with SPSS version 26.0 (IBM SPSS Statistics for Windows, Version 26.0. Armonk, NY, USA) and GraphPad Prism 9.0 (GraphPad Software Version 9.0.1 for Mac IOs. GraphPad Software, San Diego, CA, USA).

## 3. Results

A total of 474 patients with acute stroke to LVO were treated with mechanical thrombectomy in our comprehensive regional stroke center between April 2016 and January 2020 and screened for eligibility (Figure 1, Consort diagram). Here, 77 patients met inclusion criteria, while in 7 patients 90 day follow-up (FU) was missing.

In total, 70 patients were enrolled in this study. The details are presented in Table 1. Here, 82.9% (*n* = 58) suffered from the middle cerebral artery occlusion and 17.1% (*n* = 12) showed occlusion of the carotid T. In 52.9% (*n* = 37) the left hemisphere was affected. Complete reperfusion (mTICI 3) of the occluded vessel could be accomplished in 41.4% (*n* = 29), while mTICI 2b-c was achieved in a further 58.6% (*n* = 41) of cases.

Here, 32.9% (*n* = 23) patients showed an excellent clinical outcome (mRS 0–1) after 90 days, while 51.4% (*n* = 36) patients showed a good clinical outcome (mRS 0–2). Significant decreases in NIHSS could be detected over the time period from admission (NIHSS median 14, IQR 10–18) to discharge (NIHSS median 2, IQR 0–8; z = 5.8, *p* ≤ 0.001).

### 3.1. Impacts of Cortical Lesions

Regarding subgroups, 40 patients were assigned to group A with no or minimal cortical infarction and 30 patients were assigned to group B with additional involvement of eloquent areas or ≥2 non-eloquent cortical ASPECTS area. Groups did not differ with respect to their comorbidities, occlusion side, risk factors, TICI, or reperfusion time (see Table 1). Group A patients showed better excellent outcome (mRS 0–1) results at 3 months than group B (A 45% vs. B 16.7%, χ²(1) = 6.24, *p* = 0.013). In terms of good outcome (mRS 0–2) results, the groups showed no significant difference (A 55% vs. B 46.7%, χ²(1) = 0.31, *p* = 0.581). The median mRS values of the groups differed both at discharge (z = 2.5, *p* = 0.013) and at 90 days (z = 2.1, *p* = 0.021). Group A showed a significantly better median NIHSS at admission (z = 2.78, *p* = 0.005) as well as at 24 h after onset (z = 3.23, *p* = 0.001) and at discharge (z = 3.06, *p* = 0.002) compared to group B.

**Figure 3:**
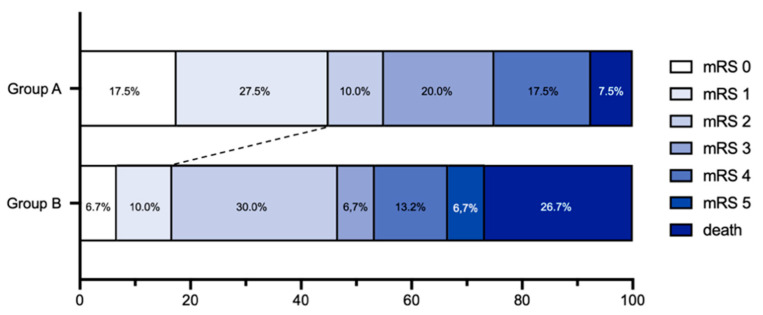
The modified Rankin Scale (mRS) score at 90 days demonstrates a shift toward excellent outcome (mRS 0–1) for patients in group A (*n* = 40) as compared with group B (*n* = 30).

Group assignment to group A or B had no significant impact on good clinical outcome (mRS 0–2; *p* = 0.491, OR = 0.716, CI [0.277–1.851]), whereas a significant impact on excellent clinical outcome was found in group A (*p* = 0.016, OR = 0.244, CI [0.078–0.768]). Hence, patients with minimal cortical infarction in the postinterventional imaging had a 75.6% better chance of excellent outcome. Group dependency could also be shown for a better mRS using ordinal logistic regression (*p* = 0.020, OR = 0.360, CI [0.150–0.842]) (see Figure 3).

We did not find a significant influence of preinterventional infarction in the ASPECTS areas C, L, or IC, neither on good (*p* = 0.532, OR = 1.139, CI [0.496–3.890]) nor on excellent outcome (*p* = 0.542, OR = 1.395, CI [0.478–4.066]). Neither on good (*p* = 0.532, OR 1.389, CI [0.496–3.890]) nor on excellent outcome (*p* = 0.111, OR 2.292, CI [0.827–6.358]) could a significant stroke side influence be confirmed.

### 3.2. ASPECTS Score

In 34 (48.6%) of patients the preinterventional ASPECTS calculation was based on cerebral magnetic resonance imaging (cMRI), while for another 36 (51.4%) patients it was based on cranial computer tomography (CT). Postinterventional ASPECTS values were determined using CT in 66 (94.3%) and MRI in 4 (5.7%) cases. In 21 patients (30.0%), CWM and internal capsule were preinterventionally not affected and infarct demarcation was only seen on postinterventional images. Overall, there was a significant worsening in ASPECTS after EVT (z = −5.4, *p* ≤ 0.001). In 44 patients (62.9%), the median ASPECTS worsened by 2 points (IQR 1-3) with intervention. In 7 patients the median ASPECTS improved by 1 point (IQR 1-1), while in 19 patients it remained the same.

### 3.3. Safety Outcome

Overall, 11 patients died within 90 days (mortality 15.7%). There was a significant difference between group A and group B (χ²(1) = 4.76, *p* = 0.029). Another 23 patients (32.9%) had an unfavorable outcome (mRS 3–5), while 3 patients (4.3%) showed postinterventional symptomatic hemorrhage. Outcome data are depicted in Table 2.

## 4. Discussion

In this explorative retrospective study, we analyzed patients who underwent EVT after LVO and showed infarct demarcations in the region of the corticospinal tracts upon postinterventional imaging. Our study indicates a good clinical outcome (mRS 0–2 after 90 days in 51.4% of the patients), which is comparable to the results of other thrombectomy trials (HERMES) [18]. Our results do not support the hypothesis that involvement of the corticospinal tracts is a poor outcome predictor, as this is not supported by our data.

Even with patient selection according to postinterventional CT or MRI controls that show the final extent of infarcts more reliably, we did not observe increased rates of poor clinical outcomes (mRS 3–5) or deaths. Our patient cohort did not differ relevantly from the usual patient collectives used for EVT studies regarding the sex profile, cardiovascular risk factors, or distribution of intracranial occlusion locations [18], although it must be added that a direct comparison was naturally not possible due to the different study designs and inclusion criteria. In contrast to previous studies, we could not detect right hemispheric stroke as a predictor for an unfavorable outcome [19]; more specifically, the outcome of our cohort was not dependent on the stroke side, as demonstrated in earlier work [20].

Although important regions related to the corticospinal tract were affected, 51.4% of the patients were functionally independent (mRS 0–2) after three months, 32.9% of patients even had an excellent clinical outcome (mRS 0–1), and 58.6% lived at home without nursing service after 90 days. This suggests that despite infarcts of these functional relevant white matter tracts, endovascular thrombectomy remains a highly effective procedure that could allow patients with LVO to live as barrier-free as possible.

In contrast to a recent evaluation that found that corticospinal tract involvement was highly predictive of poor outcomes and that infarct confinement to the gray matter was associated with good outcomes [21], we found a worse prognosis for patients with additional infarction in the cortical gray matter. Patients were already more severely affected on admission and also showed significantly worse NIHSS than group A at 24 h and at discharge. Beneath the possibility that damage of the corticospinal tracts was incomplete, the subgroup analysis of our study underlines the importance of cortical infarcts. Especially lesions in eloquent areas of the MCA territory had a significant influence on clinical outcome [22].

For the clinical outcome of patients with predominant infarcts in the course of the corticospinal tracts, research concepts of white matter plasticity may also play an important role [23].

Hypodensity upon CT and signal changes on MRI in the course of the corticospinal tracts may not be good markers for the degree and extent of white matter damage. Incomplete fiber damage with the potential for functional recovery could be missed. Other more refined imaging techniques such as diffusion tensor imaging (DTI) and tractography may provide a more detailed assessment of the white matter tract integrity [10,24,25]. Currently, DTI is not sufficiently validated for routine clinical use. Successful recanalization of the A1 and M1 segements and the ascending perforating arteries may also improve the perfusion in the vascular border zone in the central white matter adjacent to the basal ganglia and may increase the amount of salvageable brain tissue. Perfusion studies could be helpful to define the extent of the infarct core and changes after reperfusion [26]. Furthermore, our imaging analysis did not consider the functional topography of corticospinal tracts with its fan-shaped convergence from the CWM to the posterior limb of the internal capsule.

### Limitations

This retrospective single-center study has several limitations, including its small sample size and the lack of a control group (for example IVT-only). The major limitation of this study is the lack of comparison of the population with a control group without the involvement of CST. Patients with carotid-T and M1 occlusions frequently show infarcts in the territory of perforating end arteries supplying the basal ganglia. Routine postinterventional CT controls are not sensitive enough to exclude infarcts in the adjacent white matter. Furthermore, cortical infarcts without CST involvement may preferably occur in other subgroups of patients with occlusions of the proximal M2 segments or proximal M1 occlusions sparing the origin of the lenticulostriate arteries in the distal M1 segment. A controlled study was beyond the scope of our exploratory approach and would demand a larger sample of patients and the systematic use of MR-based imaging, which should be a part of future prospective studies.

One other argument could be that predictors of neurologic outcome cannot be calculated from postinterventional imaging. We selected our investigated collective based on postinterventional infarction patterns because infarct demarcation on CTs, especially in acute strokes, is not yet adequately detectable preinterventionally. The use of non-contrast computed tomography as the main pre- and postinterventional imaging modality [27] may also have an important influence. It remains unclear whether systematic use of CT perfusion or MRI would improve the correlation between the infarct topography and clinical outcome [28]. In the acute phase, the extent of DWI changes might exaggerate the final infarct volume. Systematic MRI studies including new quantitative methods for tissue evaluation may further improve the imaging prediction of functional impairment. Our study also does not reflect the functional changes gained during the rehabilitation process. Subgroup analyses were generally confined to small cohorts that introduce uncertainty to the presented effects (as indicated by the relatively wide confidence intervals). The calculation of infarct volumes and more detailed analysis of the functionality of the involved fiber tracts may increase the meaningfulness of future studies. Last but not least, the collateral status, which was not incorporated in the presented study, may also modulate patient outcomes.

## 5. Conclusions

In our pilot study, we found no indication to support the hypothesis that infarcts in the course of the corticospinal tracts predict poor clinical outcome after successful thrombectomy in patients with embolic carotid-T or M1 occlusions. In our analysis, a significant number of patients showed a favorable 90 day outcome. Additional cortical infarcts may have a greater impact on the risk of an unfavorable outcome. Further prospective studies of the relationship between the infarct topography and clinical outcome and more refined imaging tools are necessary.

## Figures and Tables

**Table 1:** Demographic data. Data are presented as numbers (%) or if marked as medians (IQR). NIHSS, National Institute of Health Stroke Scale; EVT, endovascular treatment; IVT, intravenous thrombolysis; TOAST, Trial of ORG 10172 in Acute Stroke Treatment; TICI, thrombolysis in cerebral infarction; symptom onset to reperfusion: time interval from witnessed symptom onset or last seen well to reperfusion.

	*n* (%) Overall	*n* (%) Group A	*n* (%) Group B	*p*-Value
	*n* = 70	*n* = 40	*n* = 30	
Age (y; mean ± SD)	71.8 ± 13.20	71.4 ± 13.81	72.4 ± 12.54	
Male	32 (45.7)	17 (42.5)	15 (50.0)	0.533
Secondary transferred to EVT	33 (47.1)	20 (50.0)	13 (43.3)	0.580
IVT	43 (61.4)	27 (67.5)	16 (53.3)	0.228
Length of stay (d; mean ± SD)	11.7 ± 8.69	12.5 ± 10.07	10.7 ± 6.46	0.592
**NIHSS baseline** (median, IQR)	14 (10–18)	11.5 (9–17)	16 (12–19)	0.005
**mRS baseline** (median, IQR)	0 (0–1)	0 (0–1)	0 (0–1)	0.222
**Living status prior stroke**				
Home	63 (90.0)	37 (92.5)	26 (86.7)	0.420
Nursing at home	1 (1.4)	0 (0)	1 (3.3)	0.245
Nursing home	6 (8.6)	3 (7.5)	3 (10.0)	0.712
**Risk factors**				
Smoking	10 (14.3)	6 (15.0)	4 (13.3)	0.844
Hypertension	55 (78.6)	30 (75.0)	25 (83.3)	0.400
Dyslipidemia	9 (12.9)	4 (10.0)	5 (16.7)	0.410
Diabetes	11 (15.7)	6 (15.0)	5 (16.7)	0.850
Atrial fibrillation	44 (62.9)	28 (70.0)	16 (53.3)	0.153
**TOAST**				
Large-artery atheroslerosis	6 (8.6)	2 (5.0)	4 (13.4)	0.218
Cardioembolism	46 (65.7)	28 (70.0)	18 (60.0)	0.383
Other	18 (25.7)	10 (25.0)	8 (26.6)	0.776
**Occlusion Side Left**	37 (52.9)	18 (45.0)	19 (63.3)	0.128
**Occlusion localisation**				
M1	58 (82.9)	32 (80.0)	26 (86.7)	0.464
Carotid-T	12 (17.1)	8 (20.0)	4 (13.3)	0.464
**TICI**				
2b	34 (48.6)	19 (47.5)	15 (50.0)	0.856
2c	7 (10.0)	3 (7.5)	4 (13.3)	0.421
3	29 (41.4)	18 (45.0)	11 (36.7)	0.484
Symptom onset to endovascular reperfusion (h; mean ± SD)	10.9 ± 39.71	6.2 ± 4.00	17.3 ± 60.84	0.586
Groin puncture to endovascular reperfusion (h; mean ± SD)	0.8 ± 0.48	0.8 ± 0.48	0.8 ± 0.48	0.826

**Table 2:** Data are presented as numbers (%) or if marked as medians (IQR). NIHSS, National Institute of Health Stroke Scale; mRS, modified Rankin Scale.

	*n* (%) Overall	*n* (%) Group A	*n* (%) Group B	*p*-Value
	*n* = 70	*n* = 40	*n* = 30	
**Living status discharge**				
Home	21 (30.0)	14 (35.0)	7 (23.3)	0.113
Rehab	35 (50.0)	20 (50)	15 (50.0)	1.000
Nursing home	1 (1.4)	1 (2.5)	0 (0)	0.383
Hospital	6 (8.6)	2 (5.0)	4 (13.3)	0.218
Dead	7 (10)	3 (7.5)	4 (13.3)	0.421
**Living status 90 d FU**				
Home	41 (58.6)	24 (60.0)	17 (56.7)	0.779
Nursing at home	6 (8.6)	4 (10.0)	2 (6.65)	0.622
Nursing home	5 (7.1)	4 (10.0)	1 (3.3)	0.284
Rehab	5 (7.1)	3 (7.5)	2 (6.65)	0.893
Hospital	2 (2.9)	2 (5.0)	0 (0)	0.214
Dead	11 (15.7)	3 (7.5)	8 (26.7)	0.029
**NIHSS (median, IQR)**				
After 24 h	8 (3–15)	4.5 (3–10)	13 (7–21)	0.001
At discharge	2 (0–8)	1 (0–5)	5.5 (2–12)	0.002
**mRS (median, IQR)**				
At discharge	2.5 (1–4)	1.5 (1–4)	4 (2–5)	0.013
After 90 d	2 (1–4)	2 (1–4)	3 (2–6)	0.021
**mRS outcome after 90 d**				
Good outcome (mRS 0–2)	36 (51.4)	22 (55.0)	14 (46.7)	0.581
Excellent outcome (mRS 0–1)	23 (32.9)	18 (45.0)	5 (16.7)	0.013
**Symptomatic intracranial haemorrhage**	3 (4.3)	0 (0)	3 (10.0)	0.052

## Data Availability

The data that support the findings of this study are available from the corresponding author upon reasonable request.

## Abbreviations

MCA, middle cerebral artery; ASPECTS, Alberta Stroke Program Early Computed Tomography Score; CCI, Charlson comorbidity index; CI, confidence interval; CT, computer tomography, CWT, central white matter; DTI, diffusion tensor imaging; EVT, endovascular thrombectomy; ICA, internal carotid artery; IQR, interquartile ranges; IVT, intravenous thrombolysis; LOT, large-vessel occlusion; MCA, middle cerebral artery; MRI, magnetic resonance imaging; mRS, modified Rankin Scale; SD, standard deviation; mTICI, Modified Treatment in Cerebral Infarction.
